# Effect of bombesin receptor subtype-3 and its synthetic agonist on signaling, glucose transport and metabolism in myocytes from patients with obesity and type 2 diabetes

**DOI:** 10.3892/ijmm.2015.2090

**Published:** 2015-02-04

**Authors:** NIEVES GONZÁLEZ, ANTONIO MARTÍN-DUCE, FÉLIX MARTÍNEZ-ARRIETA, ZAIDA MORENO-VILLEGAS, SERGIO PORTAL-NÚÑEZ, RAÚL SANZ, JESÚS EGIDO

**Affiliations:** 1 Renal, Vascular and Diabetes Research Laboratory, IIS-Jiménez Díaz Foundation, The Autonomous University of Madrid, Spanish Biomedical Research Network in Diabetes and Associated Metabolic Disorders (CIBERDEM), Madrid, Spain; 2 Department of Nursery, Unit of Surgery, Alcalá University, Madrid, Spain; 3 Department of General Surgery, Puerta de Hierro-Majadahonda University Hospital, The Autonomous University of Madrid, Madrid, Spain; 4 Renal, Vascular and Diabetes Research Laboratory, IIS-Jiménez Díaz Foundation, The Autonomous University of Madrid, Madrid, Spain; 5 Department of Bone and Mineral Metabolism, IIS-Jiménez Díaz Foundation, Cooperative Research Thematic Network on Aging and Frailty (RETICEF), Madrid, Spain; 6 Department of Neurology, IIS-Jiménez Díaz Foundation, Madrid, Spain

**Keywords:** bombesin receptor subtype-3, human muscle, diabetes, obesity

## Abstract

Bombesin receptor subtype-3 (BRS-3) is an orphan G-protein-coupled receptor (GPCR) member of the bombesin receptor family. Several studies have suggested an association between obesity, alterations in glucose metabolism, diabetes and the BRS-3 receptor. In this study, we focused on patients simultaneously diagnosed with obesity and type 2 diabetes (OB/T2D). The analysis of BRS-3 expression in the skeletal muscle of these patients revealed a marked decrease in the expression of BRS-3 at the mRNA (23.6±1.3-fold downregulation, p<0.0001) and protein level (49±7% decrease, p<0.05) compared to the normal patients (no obesity and diabetes). Moreover, in cultured primary myocytes from patients with OB/T2D, the synthetic BRS-3 agonist, [D-Try^6^,β-Ala^11^,Phe^13^,Nle^14^]bombesin_6–14_, significantly increased the phosphorylation levels of mitogen-activated protein kinase (MAPK), p90RSK1, protein kinase B (PKB) and p70s6K. Specifically, the ligand at 10^−11^ M induced the maximal phosphorylation of MAPKs (p42, 159±15% of the control; p44, 166±11% of the control; p<0.0001) and p90RSK1 (148±2% of the control, p<0.0001). The basal phosphorylation levels of all kinases were reduced (p<0.05) in the patients with OB/T2D compared to the normal patients. Furthermore, the BRS-3 agonist stimulated glucose transport, which was already detected at 10^−12^ M (133±9% of the control), reached maximal levels at 10^−11^ M (160±9%, p<0.0001) and was maintained at up to 10^−8^ M (overall mean, 153±7%; p<0.007). This effect was less promiment than that attained with 10^−8^ M insulin (202±9%, p=0.009). The effect of the agonist on glycogen synthase *a* activity achieved the maximum effect at 10^−11^ M (165±16% of the control; p<0.0001), which did not differ from that observed with higher concentrations of the agonist. These results suggest that muscle cells isolated from patients with OB/T2D have extremely high sensitivity to the synthetic ligand, and the effects are particularly observed on MAPK and p90RSK1 phosphorylation, as well as glucose uptake. Moreover, our data indicate that BRS-3 may prove to be useful as a potential therapeutic target for the treatment of patients with OB/T2D.

## Introduction

Bombesin receptor subtype-3 (BRS-3) is a G-protein-coupled receptor (GPCR), a member of the bombesin receptor family, which is present in both the central nervous system and the peripheral tissues, including muscle (1–5). It is frequently overexpressed in various types of tumor tissue (1,6,7), thus playing an important role in lung tumor invasiveness (8).

Moreover, the BRS-3 receptor seems to be associated with glucose homeostasis. Several studies on adipose tissue and animal models (2,9–12) have suggested an association between obesity, alterations in glucose metabolism and the BRS-3 receptor. Of note, in BRS-3 knockout (KO) mice, this receptor has been implicated in insulin release (11), as well as in the development of metabolic defects and obesity (2). Additionally, a recent study reported that the BRS-3 receptor in humans is involved in the regulation of glucose-stimulated insulin secretion (13). Moreover, human skeletal muscle expresses functional BRS-3, and lower than normal BRS-3 mRNA/protein levels have been detected in patients with obesity and type 2 diabetes (OB/T2D) (5). All the aforementioned data suggest an association between BRS-3 and human metabolic alterations (5).

BRS-3 has a low affinity for all naturally occurring bombesin-related peptides, as well as most synthetic bombesin-family member analogues (8,14). BRS-3 is an orphan receptor, whose natural ligand has not yet been described, although numerous studies on the synthetic agonists of BRS-3 have been carried out (9,13,15,16). Specifically, studies on Bag-1 and MK-5046 have demonstrated that these compounds stimulate an increase in glucose-mediated insulin secretion in insulinoma cell lines (INS-1 832/3 and MIN6), and *in vivo*, they also exert several anti-obesity effects (9,13,15,16). In BALB/3T3 cells overexpressing the human BRS-3 receptor, MK-5046 and the synthetic bombesin analogue, [D-Tyr^6^,β-Ala^11^,Phe^13^,Nle^14^] bombesin_6–14_, have shown high affinity for the receptor, and both compounds stimulate kinases involed in insulin signaling, such as p125^FAK^, phosphorylated AKT (pAKT) and mitogen-activated protein kinase (MAPK) phosphorylation (17). Moreover, in cultured primary myocytes from normal subjects, obese patients, or patients with T2D, where BRS-3 is natively expressed, the insulin-mimetic effects of [D-Tyr^6^,β-Ala^11^,Phe^13^,Nle^14^] bombesin_6–14_ have been reported, which involve an increase in kinase phosphorylation [MAPKs, p90RSK1, protein kinase B (PKB) and p70s6K], thus improving glucose metabolism, with cells from obese patients and patients with T2D being more sensitive to the ligand than cells from normal subjects (18).

BRS-3 seems to play a role in glucose metabolism, not only in normal subjects, but also in obese patients and patients with T2D. Furthermore, its ligand is capable of improving glucose parameters through common insulin signaling pathways. In the current study, we aimed to assess the following: i) the status of BRS-3 in skeletal muscle tissue from patients simultaneously diagnosed with OB/T2D, ii) the effects of [D-Tyr^6^-β-Ala^11^,Phe^13^,Nle^14^] bombesin_6–14_ on glucose-related processes in primary cultured myocytes, and iii) the role of BRS-3 and its synthetic agonist, as well as the potential therapeutic application of the synthetic agonist for patients with OB/T2D.

## Materials and methods

### Reagents

[D-Tyr^6^-β-Ala^11^,Phe^13^,Nle^14^]bombesin_6–14_ was obtained from Anaspec (Fremont, CA, USA); pork-insulin was from Novo BioLabs (Bagsvaerd, Denmark); the RNeasy Fibrous Tissue kit was purchased from Qiagen (Valencia, CA, USA); HAM’S-F-10, M-199 and Dulbecco’s modified Eagle’s medium (DMEM) were obtained from (Biochrom KG, Berlin, Germany); ethylenediaminetetraacetic acid (EDTA), rat-collagen, α-tubulin antibody (T 5168) and primers were from Sigma Chemical Co. (St. Louis, MO, USA); UDP-^14^C-glucose was from American Radiolabeled Chemicals, Inc. [(ARC), St. Louis, MO, USA]; cytochalasin-B and 2-deoxy-D-[1,2-^3^H(N)] glucose were from Hartmann-Analytic (Braunschweig, Germany); Ultima Gold scintillation liquid was obtained from Packard, (Gröninger, The Netherlands); anti-BRS-3 (PA5-26484) central region of human BRS-3 was from Pierce Biotechnology (Rockford, IL, USA); horseradish peroxidase-conjugated donkey anti-rabbit immunoglobulin (NA934), rainbow markers, the ECL western blotting kit and Hyperfilm-ECL were obtained from Amersham Biosciencies (Buckinghamshire, UK); the rabbit anti-total/anti-phosphorylated-form of p44/42MAPK (#9102/#9101), p70s6K (#9202/#9204) and PKB (#9272/#9271S) were from Cell Signaling Technology - New England Biolabs (Beverly, MA, USA); the phosporylated form of p90RSK1 (ref. 01–418) was from Millipore (Temecula, CA, USA), the high-capacity cDNA reverse transcription kit was from Applied Biosystems (Cheshire, UK) and Premix Ex Taq™ was from Takara Clontech (Mountain View, CA, USA). All other commonly used chemicals were from Sigma or Merck (Merck Pharma Quimica, S.A., Barcelona, Spain).

### Biological materials

Human skeletal muscle samples were obtained after informed consent from normal subjects without any alterations in glucose metabolism, as indicated by normal levels of glucose and glycated hemoglobin (hemoglobin A1c; HbA1c), as well as from patients with OB/T2D (Table I) undergoing surgery for well-established clinical conditions [surgery in normal subjects was for conditions such as subtotal thyroidectomy) and bariatric surgery for patients with OB/T2D]. The patients underwent surgery for purposes not related to this study. This study was approved by the Ethics Committee of the IIS-Jiménez Díaz Foundation, Madrid, Spain, in accordance with the ethical principles stated in the Declaration of Helsinki. The samples were cleaned with HAM’s F-10, dissected from the connecting tissue and divided into sections of 120 and 400 mg. The first set was stored at −80°C for the extraction of total RNA. However, in the 400 mg sections, the isolation of myoblasts and posterior culture for growth and differentiation were performed inmmediately following isolation as previously described (19). In the OB/T2D group, all patients were prescribed with oral antidiabetic drugs (Table I). The normal subjects did not receive glucose-related drugs. In all cases, the isolated myoblasts were grown for 6 weeks in 5.5 mM glucose and insulin-free medium. No differences in the growth rate or cell morphology were observed (data not shown).

### Isolation of total RNA and reverse transcrtipion-quantitative (real-time) PCR (RT-qPCR)

Total RNA was isolated from the sections of human skeletal muscle tissue using the RNeasy Fibrous Tissue kit, according to the manufacturer’s instructions. For RT-qPCR, cDNA was synthesized from 3 *μ*g (BRS-3) of total RNA using the High Capacity cDNA Reverse Transcription kit. The samples from the skeletal muscle sections were subjected to quantitative amplification using the primer sets for human *BRS-3* (5′-GGCAGTTGTGAAGCCACTTGA-3′ and 5′-AGACGCAGCCAGCTTTTACAC-3′), Premix Ex Taq™ and the StepOne Real-Time PCR system (Applied Biosystems). The conditions for amplification and detection (2 min at 95°C; 50 cycles of 15 sec at 95°C; 30 sec at 60°C) were optimized, and gene expression was normalized to that of the housekeeping *18S* gene. The mRNA copy numbers were calculated for each sample using the cycle threshold (Ct) value, and the results were expressed as n-fold mRNA values as previously reported by Livak and Schmittgen (20).

### Immunoblotting

Tissue samples (120 mg) were homogenized at 4°C in 1,25% Triton X-100 containing 250 mM sucrose, 20 mM Tris/HCl, 2·5 mM MgCl_2_, 50 mM β-mercaptoethanol, 1,2 mM EGTA, 1 mM Na_3_VO_4_, 5 mM Na_4_P_2_O_7_, 50 mM NaF, 2 *μ*M leupeptin, 2 *μ*M pepstatin, pH 7.4 and 2 mM phenylmethylsulfonyl fluoride, then maintained at 4°C for 30 min, and finally centrifuged at 15,000 × g. The supernatant (tissue lysate), containing cytosolic and solubilized membranes, was kept at −70°C for performing the BRS-3 protein assays.

Equal amounts of tissue lysates from each muscle sample were subjected to sodium dodecyl sulfate-polyacrylamide gel electrophoresis (SDS-PAGE) (21), in parallel with molecular weight markers, on an 8% resolving gel. The separated proteins were then transferred onto a nitrocellulose membrane in a semi-dry system (Trans-Blot SD Semi-dry Transfer Cell; Bio-Rad, Hercules, CA, USA). For immunodetection, a western blotting kit was used following the manufacturer’s instructions (Amersham Biosciencies), using total and phosphorylated (PKB, p70s6K and MAPKs) antibodies. In addition, p90RSK1 phosphorylated antibody, as well as BRS-3 antibody and α-tubulin antibody (internal loading control) were used. As the secondary antibody, horseradish peroxidase-conjugated donkey anti-rabbit immunoglobulin was used, and detection was carried out by enhanced chemiluminescence and quantification by autoradiography and densitometry, as previously described (22).

The densitometric measurements of the phosphorylated protein kinase or BRS-3 protein were normalized (percentage phosphorylated/total kinase or α-tubulin) to the value obtained from the myocytes incubated in the absence of the peptide which was used as the control value.

### Glucose transport

The myocytes were treated as previously described in the study by González *et al* (19). Briefly, the cells were pre-incubated for 30 min at 37°C without (control) or with [D-Tyr^6^,β-Ala^11^,Phe^13^,Nle^14^]bombesin_6–14_ (10^−12^ to 10^−7^ M) or insulin (10^−8^ M), followed by 5 min of incubation at 37°C in the additional presence of 0.2 *μ*Ci (6.5 pmol) 2-deoxy-D-[1,2-^3^H(N)]glucose. The myocytes, after being washed for the removal of radioactivity incorporated into the cells, were scraped, dissolved in 1 ml 1 N NaOH and were then added to 3 ml scintillation liquid for β-counting. The total glucose content was corrected by the unspecific glucose uptake value, obtained in some of the cell samples from each experiment treated in parallel with 0.1 mM cytochalasin B.

### Glycogen synthase a (GSa) activity

GS*a* activity was examined in cells incubated in DMEM, at 37°C for 10 min, in the absence (control) or presence of BRS-3 agonist or insulin. The myocytes were immediately homogenized (FNa 100 mM, glycogen 0.5% w/v, glycylglycine 50 mM, EDTA 35 mM, pH 7.4) and frozen at 160 *μ*g protein/40 *μ*l until the enzymatic activities were assayed. A total of 80 *μ*l of [UDP-glucose 0.375 mM, glycogen 1.5% w/v, glycylglycine 90 mM, Na_2_SO_4_ 15 mM, UDP-^14^C-glucose 0.375 *μ*M (1.25 *μ*C/ml), pH 7.4] and 40 *μ*l of homogenized cells were incubated at 20°C for 15 min. The reaction was terminated by the addition of 200 *μ*l potassium hydroxide (KOH) (0.5 N) at 4°C. The final glycogen extraction was carried out as previously described in the study by Fleig *et al* (23).

### Statistical analysis

The results are expressed as the means ± SEM, together with the number of observations (n). The statistical significance (p<0.05) of the increments was assessed by one-way analysis of variance (ANOVA), according to Levene’s test, all variances were homogeneous, followed by the post-hoc Bonferroni test, using the Statistical Package for Social Sciences (SPSS 21.0).

## Results

### BRS-3 expression

In order to evaluate the potential role of the BRS-3 receptor in glucose homeostasis, we investigated the BRS-3 gene/protein expression levels in the skeletal muscle sections obtained from the patients with OB/T2D, and compared these levels to those of the normal patients.

**Figure 1:**
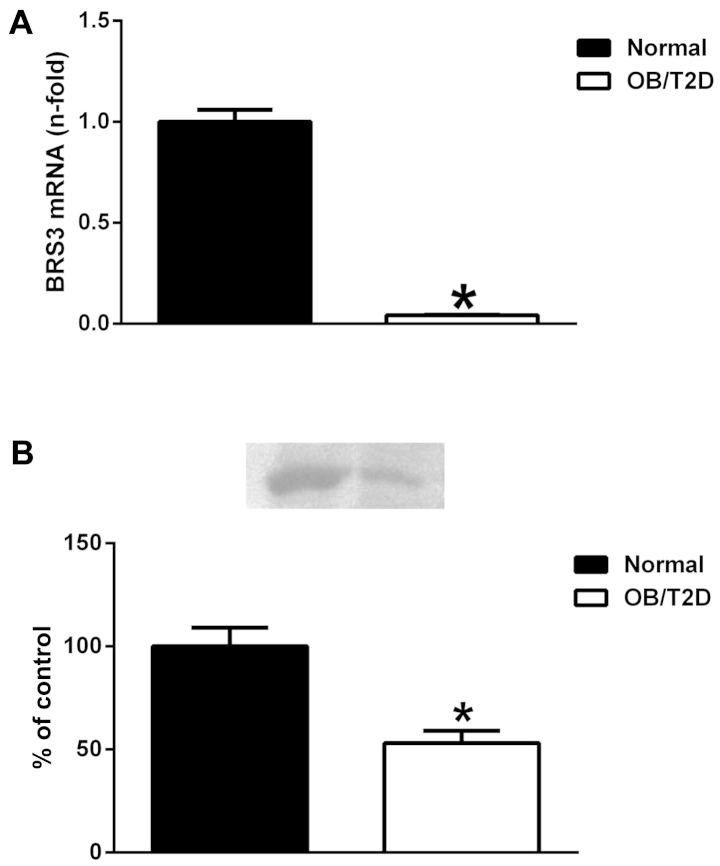
Bombesin receptor subtype-3 (BRS-3) gene expression and protein levels in muscle tissue sections from patients with obesity and type 2 diabetes (OB/T2D) and normal subjects. (A) BRS-3 gene expression level in skeletal muscle tissue sections. Values (means ± SEM) are expressed as copy of numbers by using the Ct value relative to that of the normal subjects after normalization against 18S rRNA (2^−ΔΔCt^). Patients with OB/T2D, n=5 and normal subjects, n=19. ^*^p<0.001, OB/T2D vs. normal subjects. (B) BRS-3 protein expression level in skeletal muscle tissue sections. Values (means ± SEM), corresponding to total protein extracted are expressed as a percentage of measurements relative to the normal subjects. Patients with OB/T2D, n=5 and normal subjects, n=4; upper panel corresponds to a representative immunoblot, and lower panel represents quantitative analysis of the BRS-3 protein level. ^*^p<0.001 OB/T2D vs. normal subjects.

As shown in Fig. 1A, In 5 patients with OB/T2D, the BRS-3 mRNA levels were significantly lower (23.6±1.3-fold downregulation, p<0.001) compared to the normal subjects (n=19). These results are in accordance with those obtained for BRS-3 protein expression (Fig. 1B); a significant decrease was observed in the protein expression levels of BRS-3 in the patients with OB/T2D (n=3), compared to 3 normal subjects.

### Cell signaling

**Figure 2:**
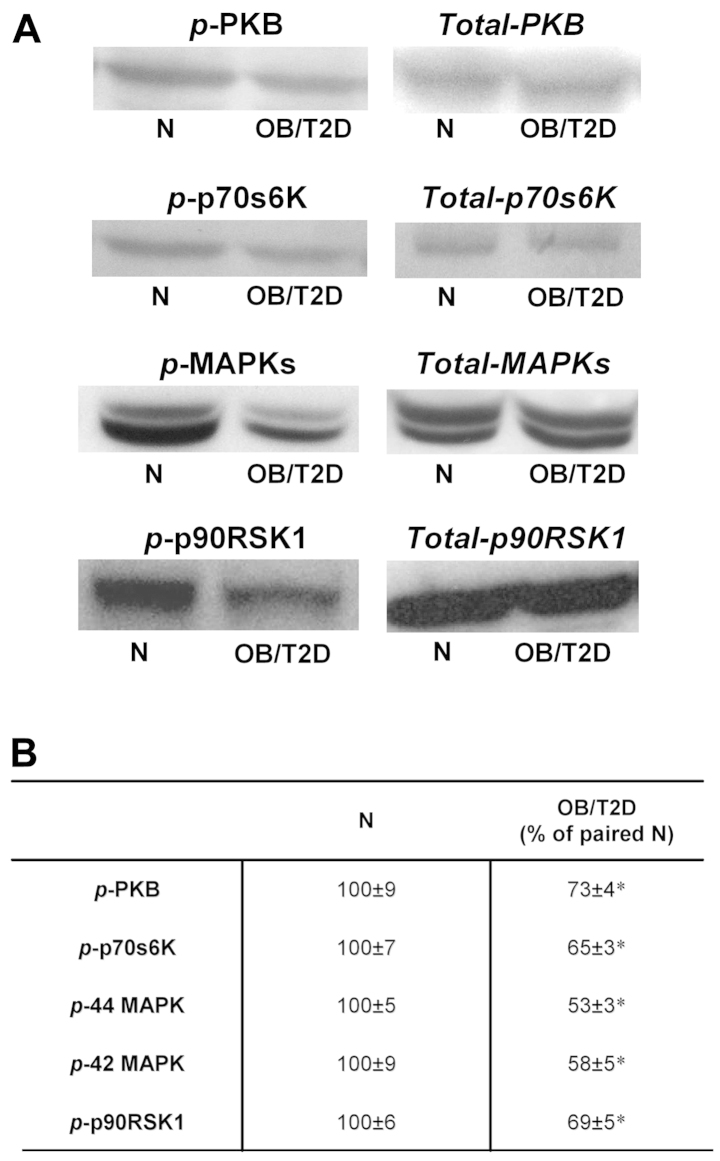
PKB, p70s6K, p42/p44 MAPKs and p90RSK1 phosphorylated forms, in cultured primary myocytes from patients with obesity and and type 2 diabetes (OB/T2D) compared to normal subjects (N). Results (means ± SEM) are expressed relative to the control value obtained from cells from normal subjects. Patients with OB/T2D, n=5 and normal sujbects, n=2; (A) representative immunoblots, and (B) quantitative analysis. ^*^p<0.001 OB/T2D vs. normal sujbects.

When cultured myocytes from patients with OB/T2D (n=5) were examined in parallel with those from the normal subjects (n=2), the basal phosphorylation levels of PKB, p70s6K, p42/p44 MAPKs and p90RSK1 were significantly lower (p<0.001) in the cells from patients with OB/T2D compared to those from the normal subjects (Fig. 2).

**Figure 3:**
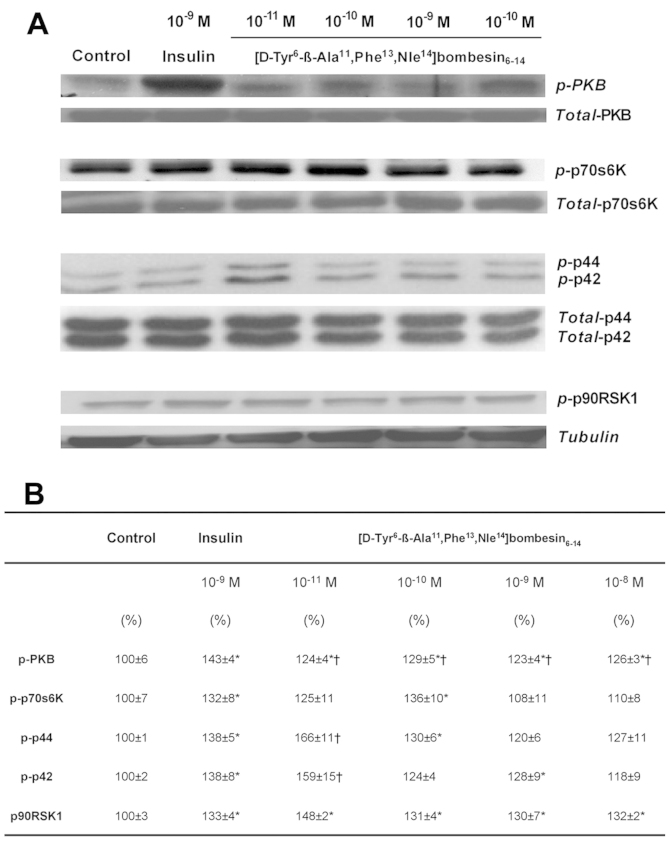
Effect of treatment with 10^−11^ to 10^−8^ M [D-Tyr^6^-β-Ala^11^,Phe^13^,Nle^14^] bombesin_6–14_ and insulin on PKB, p70s6K, p42/p44 MAPK and p90RSK1 phosphorylation in cultured primary human myocytes from patients with obesity and type 2 diabetes (OB/T2D) incubated for 3 min. (A) Representative immunoblots. (B) Quantitative analysis of enzyme phosphorylation levels; values (means ± SEM) are relative to the respective paired control obtained form cellular samples incubated in the absence of the peptide corresponding to patients with OB/T2D (n=5). ^*^p<0.02 vs. control; ^†^p<0.0001 vs. treatment with insulin.

In order to determine the signaling pathways involved in the activation of the BRS-3 receptor, we investigated the ability of the BRS-3 agonist to interact with this receptor, using a primary myocyte culture from 5 patients with OB/T2D. The results obtained for 4 kinases examined in the present study revealed that in the patients with OB/T2D, the increased phosphorylation of these enzymes was observed in response to treatment with [D-Tyr^6^-β-Ala^11^,Phe^13^,Nle^14^]bombesin_6–14_. The presence of insulin also stimulated the activity of PKB, p70s6K, p42/p44 MAPKs and p90RSK1. Specifically, treatment with the BRS-3 agonist at 10^−11^ to 10^−8^ M slightly increased the PKB phosphorylation levels (overall mean, 126±2% of control; p<0.02)l; these levels were lower (p<0.0001) than those obtained by treatment with 10^−9^ M insulin (Fig. 3). Distinctively, the activation of p70s6K induced by treatment with 10^−10^ M ligand (p=0.029), did not differ from that obtained by treatment with 10^−9^ M insulin (Fig. 3). When higher concentrations of [D-Tyr^6^-β-Ala^11^,Phe^13^,Nle^14^]bombesin_6–14_ were used (10^−9^–10^−8^ M), no significant increments were detected (overall mean, 109±4% of control) compared to the basal values (Fig. 3). However, when cells from patients with OB/T2D were incubated in the presence of [D-Tyr^6^-β-Ala^11^,Phe^13^,Nle^14^]bombesin_6–14_, the expression pattern of the p42/p44 MAPKs differd (Fig. 3). The expression of both enzymes achieved the maximal increment following treatment with 10^−11^ of the compound; however, this expression decreased following treatment with higher concentrations of the ligand (10^−10^ and 10^−9^ M), although the expression was maintained at a higher level compared to the basal level (Fig. 3). Moreover, the stimulatory effects induced by insulin at 10^−9^ M on p44 MAPK phosphorylation were significantly less prominent compared to the effects induced by treatment with 10^−11^ M agonist (Fig. 3). As regards the p90RSK1 phosphorylation levels (Fig. 3), a relevant increase was observed following treatment with 10^−11^ M of the BRS-3 ligand (148±2% of the control, p<0.0001); the p90RSK1 phosphorylation levels were at similar levels following treatment with 10^−10^ to 10^−8^ M of the agonist and 10^−9^ M insulin (Fig. 3).

### Glucose transport and GSa activity

Glucose transport and GS*a* activity are essential parameters of glucose homeostasis in muscle tissue. Due to their relevance, we examined the effects of the BRS-3 agonist on glucose transport and metabolism in cultured primary human myocytes from 4 patients with OB/T2D (Table II).

The presence of the BRS-3 ligand elicited a stimulation of glucose transport [with respect to the value obtained in the cells incubated in the absence of the peptide (15.7±2.4 fmol/2×10^4^ cells, n=4)], which was almost significant (p=0.082) following treatment with the ligand at 10^−12^ M (20.8±1.4 fmol/2×10^4^ cells, n=4), reached a maximal level at 10^−11^ M (25.1±1.4 fmol/2×10^4^ cells, n=4, p<0.001), and was thereafter maintained up to the dose of 10^−8^ M (overall mean value: 23.7±1.1 fmol/2×10^4^ cells, n=4, p<0.008), and then slightly decreased by treatment at 10^−7^ M (20.4±1.1 fmol/2×10^4^ cells, n=4). Despite the fact that the maximal stimulatory effect of the BRS-3 agonist on glucose transport was produced by treatment with 10^−11^ M of the compound, treatment with 10^−9^ M insulin (Table II), achieved a significantly higher net glucose uptake (31.8±1.4 fmol/2×10^4^ cells, n=4, p=0.009 vs. treatment with 10^−11^ M BRS-3 agonist).

The ligand clearly increased GS*a* activity in the myocytes obtained from 4 patients with OB/T2D (Table II). Specifically, treatment with 10^−11^ M of the compound induced the maximal increase in enzyme activity (0.020±0.001 U/g protein, n=4, p<0.0001), compared to the value obtained in the cells incubated in the absence of peptide (0.020±0.0013 U/g protein, n=4). This effect was equal to that exerted by insulin (10^−9^ M, 0.029±0.002 U/g protein, n=4, p<0.0001 vs. control) (Table II). Although treatment with higher concentrations of the ligand (10^−10^ M, 0.031±0.002 U/g protein, n=4, p<0.0001 vs. control; 10^−9^ M, 0.028±0.002 U/g protein, n=4, p=0.0008 vs. control; 10^−8^ M, 0.028±0.002 U/g protein, n=4, p<0.0001 vs. control) produced significant increases in GS*a* activity, it did not improve the cellular response to the BRS-3 agonist (Table II).

## Discussion

Studies on the BRS-3 receptor in muscle cells from patients with OB/T2D have demonstrated its role in glucose homeostasis (13). In this regard, the BRS-3 receptor is considered a molecular target; therefore, a BRS-3 agonist with insulin-mimetic effects may be used as a therapeutic tool for these patients (5,18).

In this study, we focused on patients simultaneously diagnosed with OB/T2D, in order to determine BRS-3 expression levels in skeletal muscle sections and to assess the effects of the BRS-3 agonist, [D-Tyr^6^-β-Ala^11^,Phe^13^,Nle^14^]bombesin_6–14_, on glucose-related processes in primary cultured myocytes.

In the analysis of the 10 patients with OB/T2D included in this study, the range of variability in the clinical parameters was kept as minimal as possible between the normal subjects and the patients. The standard treatment for T2D is metformin, and cells from the 2 study groups showed an equal growth rate and morphology, as previously observed in myocytes from patients with OB/T2D compared to cells from normal subjects (18). Sample collection and classification was extremely challenging, due to the requirements in terms of consistency and homogeneity of the samples obtained from patients with OB/T2D. However, the recruitment of normal subjects based on metabolic characteristics was less complex due to the higher availability of such individuals.

The analysis of BRS-3 gene expression in muscle sections obtained from patients with OB/T2D exhibited a marked decrease in BRS-3 compared to the normal subjects. This finding is in agreement with the results from a previous study, where a decrease in the mRNA expression levels of BRS-3 in the muscle tissue of patients with metabolic conditions was detected (5). However, the expression level of BRS-3 in patients with OB/T2D reported in this study was much lower than that previously observed in patients affected by either T2D or obesity (5), thus suggesting a potential synergy in the negative impact of these 2 conditions on BRS-3 gene expression.

The analysis of BRS-3 protein levels showed some similarities and differences with previous data. Our results revealed that the BRS-3 protein expression levels in muscle tissue were significantly lower in patients with OB/T2D compared to the normal subjects. This reduction was not different to that observed in T2D patients (5); however, it was lower than the level reported in obese patients (5).

These facts underline the importance of a functional BRS-3 receptor in altered metabolic states in humans. Its significance has already been suggested by Ohki-Hamazaki *et al* (2), whose study indicated that mice lacking functional BRS-3 developed mild obesity, associated with hypertension and an impairment of glucose metabolism, a reduced metabolic rate, increased feeding efficiency and subsequent hyperphagia.

*In vitro* experiments carried out on myocytes from normal subjects and patients with obesity or T2D have demonstrated the ability of insulin to enhance BRS-3 expression (5), which may also be a possible mediator in the regulation of BRS-3 gene expression, as BRS-3 expression has been detected in cells expressing high levels of insulin receptor, such as fibroblasts, where certain regulatory processes are produced by the hormone (24,25). In muscle biopsies from patients with T2D (daily treatment with insulin prior to surgery), the decrease in the mRNA expression level of BRS-3 compared to the levels of the normal subjects was similar to the decrease detected in the muscle tissue sections from patients with obesity (5). It was also obseved that patients with OB/T2D receiving anti-diabetic drugs orally showed a maximal decrese in BRS-3 gene expression. Therefore, further studies are required to fully elucidate the effects of insulin and oral anti-diabetic drugs on BRS-3 expression in muscle biopsies from patients with OB/T2D.

Recently, the use of several BRS-3 agonists has been demonstrated to define BRS-3-signaling pathways. MK-5046 and a modified synthetic bombesin compound [D-Tyr^6^,β-Ala^11^,Phe^13^,Nle^14^]bombesin_6–14_ (17) have been shown (in a mouse fibroblast cell line transfected with human BRS-3) to increase p125^FAK^, pAKT and MAPK phosphorylation (17). Moreover, in primary myocytes isolated from normal subjects and patients with altered metabolic states (patients with obesity or T2D, where BRS-3 is natively expressed), the insulin-mimetic effects of [D-Tyr^6^,β-Ala^11^,Phe^13^,Nle^14^] bombesin_6–14_ have been observed (18). Increases in MAPK, p90RSK1, PKB and p70s6K phosphorylation were detected, with myocytes from patients with obesity and T2D being more sensitive to the ligand than those from normal subjects; cells from patients with T2D were even more sensitive than those from patients with obesity (18). Our results obtained from myocytes from patients with OB/T2D, showed a higher sensitivity of these cells to the agonist compared to cells from normal subjects. The BRS-3 agonist at 10^−11^ M induced the maximal p42/p44 MAPK and p90RSK1 phosphorylation, which was higher than that previously detected in cells from patients with T2D (18). However, in myocytes from patients with OB/T2D, although the presence of the ligand at the same concentration exerted a significant stimulatory effect on PKB and p70s6K phosphorylation, this effect did not differ to that previously observed in myocytes from patients with T2D (18). The basal phosphorylation levels of PKB, p70s6K, p42/p44 MAPKs and p90RSK1 were significantly lower in the cells from patients with OB/T2D compared to those from normal subjects, as previously observed in patients with obesity, apart from p90RSK-1, where no modification was reported (18,26). However, in cells from patients with T2D, no decrease in basal kinase activity was detected (18,27), which seems to reflect a major influence of obesity on the reduced basal level detected in patients with OB/T2D. Although it is not conclusive, this finding is in agreement with the findings from studies on adipocytes from rats with T2D, demonstrating that the basal phosphorylation levels of these kinases did not differ significantly from those detected in animals without metabolic alterations (28). This finding has also been shown in adipocytes from patients with obesity, with the respective basal activity of PKB, MAPKs, but not p70s6K, being decreased compared to that of the corresponding normal subjects (29). By contrast, it has been found that in soleus muscle samples from rats with T2D, PKB phosphorylation levels were lower than those observed in normal rats, whereas the phosphorylation levels of MAPKs and p70s6K were significantly higher (30).

Further assays were performed in cells from patients with OB/T2D in order to investigate 2 key parameters of glucose homeostasis, glucose transport and GS*a* activity. The results revealed that either variable, the glucose uptake or enzyme activity, was increased in the presence of the BRS-3 agonist. Specifically, treatment of the cells from patients with OB/T2D with 10^−12^ M of the compound induced a significant increment in glucose transport, with maximal effects observed at 10^−11^ M. This finding suggests a relevant sensitivity of cells from patients with OB/T2D to the BRS-3 ligand. Although no effects of insulin on glucose uptake in adipocytes in BRS-3-KO mice (possibly due to an impaired GLUT-4 translocation) has been observed (11), in our study, in myocytes from patients with OB/T2D, both the BRS-3 agonist and insulin affected glucose transport, as has been previously reported in myocytes from patients with either OB or T2D (5,26,27).

Our results revealed that in myocytes from patients with OB/T2D, treatment with [D-Tyr^6^,β-Ala^11^,Phe^13^,Nle^14^] bombesin_6–14_ at 10^−11^ M induced a significant increase in GS*a* activity similar to that exerted by higher concentrations of insulin (10^−9^ M), almost having a normalizing effect, as previousy observed in patients with in OB or T2D (18). The activation of GS*a* is preceded by the inhibition of glycogen synthase kinase-3 (GSK-3), which is closely related to p70s6K, p42/44 MAPKs and p90RSK1 (18,31,32). Previous studies, as well as the current study found an increase in the p90RSK1 phosphorylation levels induced by [D-Tyr^6^,β-Ala^11^,Phe^13^,Nle^14^] bombesin_6–14_ through the BRS-3 receptor (18), which seems to be related to the transcription factor, CREB (33), involved in BRS-3 activation in humans (18,34).

In conclusion, currently available information on the BRS-3 receptor (gene regulation, activation, binding or signaling pathways) is limited. However, recent studies (5,18), as well as the data from the present study, emphasize the importance of this receptor in human metabolic conditions. The novelty of the present study lies in the fact that it was conducted on skeletal muscle samples from patients being simultaneously affected by 2 major metabolic disoders, i.e. obesity and T2D. Our results further support the concept that the BRS-3 receptor and its synthetic agonist may prove to be useful as a therapeutic target in the treatment of patients with OB/T2D.

## Figures and Tables

**Table I:** Metabolic characteristics of patients with obese/type 2 diabetes (OB/T2D) and normal subjects included in the study.

	Patients with OB/T2D	Normal subjects
Female/male	3/7	19/4
Age (years)	49±3	50±3
BMI (kg/m^2^)	51±3^a^	24±1
Glucose (mg/dl)	112±6^a^	96±2
HbA1c	6.4±0.2	4.8±0.3
Cholesterol (mg/dl)	184±9	185±9
HDL (mg/dl)	48±4	56±3
LDL (mg/dl)	110±9	116±6
Tryglycerides (mg/dl)	147±18^a^	98±9
Years from diagnosis of type 2 diabetes	4.3±1.0	–
Treatment	Metformin (1,700–2,550 mg)	–

Values are the means ± SEM. Normal subjects, n=23; patients with OB/T2D patients, n=10.

a p<0.001 vs. normal subjects. BMI, basal metabolic rate; HbA1c, hemoglobin A1c; HDL, high-density lipoprotein; LDL, low-density lipoprotein.

**Table II:** Effect of [D-Try^6^,β-Ala^11^,Phe^13^,Nle^14^]bombesin_6–14_ and insulin on glucose transport and glycogen synthase *a* activity in myocytes from patients with obese/type 2 diabetes (OB/T2D).

	Control no peptide	% of paired control [D-Try^6^,β-Ala^11^,Phe^13^,Nle^14^]bombesin_6–14_						Insulin^a^
		10^−12^ M	10^−11^ M	10^−10^ M	10^−9^ M	10^−8^ M	10^−7^ M	10^−8^ M/10^−9^ M
Glucose transport	15.7±2.4 fmol/2×10^4^ cells	133±9	160±9^a^	146±12^a^	147±9^a^	167±14^a^	130±8	202±9^a^,^b^
Glycogen synthase *a*	0.020±0.001 U/g	–	165±16^a^	153±11^a^	140±10^a^	142±11^a^	–	147±9^a^

Results are the means ± SEM; n=4

a p≤0.008 vs. control;

b p=0.009 vs. treatment with 10^−11^ M [D-Try^6^,β-Ala^11^,Phe^13^,Nle^14^]bombesin_6–14_. ^a^The maximal effect on glucose transport was induced by 10^−8^ insulin; in signaling and metabolic assays (i.e., glycogen synthase *a* activity), the maximal effect induced by insulin was achieved at 10^−9^.

## Abbreviations

BRS-3: bombesin receptor subtype-3
T2D: type 2 diabetes
OB/T2D: obesity and type 2 diabetes
